# ArhGAP9, a novel MAP kinase docking protein, inhibits Erk and p38 activation through WW domain binding

**DOI:** 10.1186/1750-2187-2-1

**Published:** 2007-02-06

**Authors:** Boon K Ang, Chun Y Lim, Sharon S Koh, Neelamegam Sivakumar, Shahrizan Taib, Kim B Lim, Sohail Ahmed, Guna Rajagopal, Siew H Ong

**Affiliations:** 1 Institute of Molecular and Cell Biology, 61 Biopolis Drive, Proteos, 138673, Singapore; 2 Bioinformatics Institute, 30 Biopolis Street, Matrix, 138671, Singapore; 3 Department of Microbiology, Yong Loo Lin School of Medicine, 10 Medical Drive, National University of Singapore, 117597, Singapore; 4 Department of Physiology, Yong Loo Lin School of Medicine, 10 Medical Drive, National University of Singapore, 117597, Singapore; 5 Centre for Molecular Medicine, 61 Biopolis Drive, Proteos, 138673, Singapore

## Abstract

We have identified human ArhGAP9 as a novel MAP kinase docking protein that interacts with Erk2 and p38α through complementarily charged residues in the WW domain of ArhGAP9 and the CD domains of Erk2 and p38α. This interaction sequesters the MAP kinases in their inactive states through displacement of MAP kinase kinases targeting the same sites. While over-expression of wild type ArhGAP9 caused MAP kinase activation by the epidermal growth factor receptor (EGFR) to be suppressed and preserved the actin stress fibres in quiescent Swiss 3T3 fibroblasts, over-expression of an ArhGAP9 mutant defective in MAP kinase binding restored EGFR-induced MAP kinase activation and resulted in significant disruption of the stress fibres, consistent with the role of Erk activation in disassembly of actin stress fibres. The interaction between ArhGAP9 and the MAP kinases represents a novel mechanism of cross-talk between Rho GTPase and MAP kinase signaling.

## Background

The extensive study of the molecular mechanisms of intracellular signal transduction over the past two decades have elucidated major pathways that are important in controlling cell functions and have provided important insights in drug discovery. Two of the most well-studied pathways include the Mitogen-activated protein (MAP) kinase cascade and signaling by small-GTP binding GTPases of the Ras superfamily. These pathways are essential in normal development and aberrant functions have resulted in a wide range of pathophysiological conditions.

The MAP kinase cascades play important roles in a wide variety of cellular functions and are evolutionarily conserved. In mammals there are four subfamily members, namely, Erk1/2, p38, Jnk and Erk5. Each MAP kinase has its own activators, substrates and inactivators. The MAP kinase cascade is tightly regulated in order to achieve cellular functions that are spatially and temporally precise. Recent studies have revealed two mechanisms regulating the MAP kinase signaling, namely the docking and scaffolding interactions. MAP kinases possess a common docking (CD) domain that is localized to the C-terminal region of the kinase domain for interaction with docking proteins including upstream activating kinases MAP kinase kinases (MAPKKs), MAP kinases activating protein kinases (MAPKAPs), MAP kinase-specific phosphatases (MKPs) and MAP kinase substrates such as Elk [1]. These docking proteins all contain basic residues in the Docking (D) domain that form electrostatic interactions with acidic residues in the CD domain of the MAP kinases [1]. Scaffolding proteins interact with several components of the MAP kinase cascades to assemble the enzymes and substrates into a same complex to achieve signaling specificity [2].

The Rho GTPases are principally involved in the regulation of the actin cytoskeleton. The switch mechanism of Rho GTPases is tightly regulated through binding to GTP or GDP [1,2]. The human genome contains 22 Rho GTPases, 80 Rho Guanine nucleotide Exchange Factors (RhoGEF's) which catalyze the GDP exchange by GTP and about same number of Rho GTPase-Activating Proteins (RhoGAP's) which bind to Rho proteins to increase their rates of hydrolysis of bound GTP. The RhoGAP's serve as important negative regulators of Rho signaling [3-5].

The abundance of Rho proteins and their regulators and the discovery of mutations in a number of them in human diseases underscore the importance of Rho GTPase signaling in normal and aberrant development [6,7]. For example, studies in p190 RhoGAP knockout mice showed that the RhoGAP is required for axon outgrowth, guidance and fasciculation and neuronal morphogenesis [8,9]. Genetic aberrations in two neuronal RhoGAPs, namely oligophrenin and MeGAP/srGAP3, had been found in two distinct types of human mental retardation [10,11]. Defective Rho GTPase signaling has also been implicated in tumorigenesis and metastasis [7,12]. The RhoGAP member *DLC1 *(Deleted in Liver Cancer 1) had been shown to be deleted in a large number of primary hepatocellular carcinoma (HCC) and HCC cell with the implication of its role in the inhibition of tumorigenicity and invasiveness [13,14]. The focal adhesion kinase associated RhoGAP, *GRAF*, was identified as a fusion partner of the mixed-lineage leukemia (MLL) gene by unique chromosome translocation in juvenile myelomonocytic leukemia [15]. The fusion of *BCR *with *ABL *oncogene is a leukemia-associated chromosomal translocation which resulted in the fusion proteins p120 and p190 that lacked the RhoGAP domain of *BCR *[16].

The RhoGAP family is defined by the presence of a ~150 amino acid conserved RhoGAP domain in the primary sequences of proteins and shares at least 20% sequence homology amongst the family members [3,16]. The binding of the RhoGAP domain with GTP-bound Rho proteins accelerates the GTPase activity of the latter. One subfamily of recently discovered RhoGAP proteins consisting of ArhGAP9 and ArhGAP12 contain a diverse combination of functional protein domains, including RhoGAP, SH3, WW and PH domains [17,18]. ArhGAP9 was reported to be active towards cdc42 and Rac1 but not RhoA and to repress the adhesion of a human leukemia cell line KG-1 to fibronectin and collagen [17].

One major theme important in molecular signaling is regulated protein-protein interactions which result in the assembly of protein complexes to transmit intracellular signals, allow for control of signaling specificity and mediate cross-talk between different pathways to coordinate signaling outcomes. These regulated molecular interactions occur through defined protein domains and complementary recognition motifs on the binding partners.

The WW domain is a 35–40 amino acid protein interaction domain with two signature tryptophan residues spaced by 22 amino acids [19,20]. WW domains recognize several classes of distinct peptide ligands comprising proline-rich sequences, such as PPDY, PPLP, PPPPP, PPXPPXR, PPRXXP (X: any amino acid), PR motifs or phosphorylated threonine or serine-proline (pT/pS)-P sites. WW domains are found in many eukaryotes and are present in approximately 50 human proteins [19] and have been classified into four groups based on their binding to the different types of peptide ligands. Group I WW domains have been shown to recognize PY motifs, Groups II and III WW domains have relatively broad and overlapping specificity for ligands comprising PPLP and PR motifs, and Group IV WW domains recognize (pS/pT)-P motifs. Disruption of WW domain interactions has been implicated in cancer [21-23] and in hereditary disorders such as Duchenne muscular dystrophy [20], the Liddle syndrome of hypertension [24,25], the Rett syndrome [26], as well as in Alzheimer's [27-29] and Huntington's diseases [30,31].

We report here that human ArhGAP9 is a novel docking protein of the MAP kinases Erk2 and p38α. We show that the binding sites involved clusters of complementarily charges residues located in the common docking (CD) domain of Erk and p38 and the C-terminal region of the WW domain of ArhGAP9, and that through this interaction, cross-talk between the Rho GTPase and MAP kinase signaling pathways could occur to control actin remodeling.

## Results

### Novel interaction between Erk and p38 MAP kinases and the WW domain of the ArhGAP9 RhoGAP

**Figure 1:**
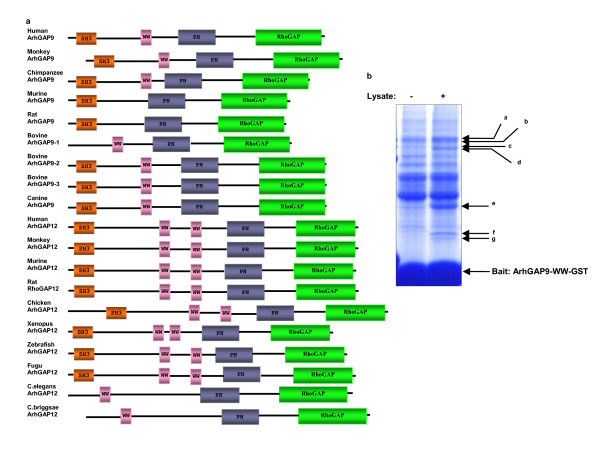
***Erk2 binds to human ArhGAP9 at the WW domain***. *a. Evolutionary conservation of ArhGAP9 and ArhGAP12*. Schematic representation of ArhGAP9 and ArhGAP12 domain structures of different species as predicted by SMART domain prediction tool. *b. Identification of Erk2 as an interacting protein of the WW domain of human ArhGAP9*. Immobilized ArhGAP9-WW-GST precipitated several proteins (bands a-g) from rat brain lysate detected by Colloidal Coomassie Blue. The bands were excised for in-gel reduction, S-alkylation and trypsin hydrolysis. The peptides were identified by mass spectrometry. Bands f and g generated 15 unique peptides covering 40% of the MAP kinase, Erk2 sequence.

ArhGAP9 and ArhGAP12 are highly similar RhoGAP proteins which possess the SH3, WW, PH and RhoGAP domains. We have cloned the full-length mouse ArhGAP9 cDNA (GeneBank accession no. DQ498128) and found that although it shares 64% sequence identity with human ArhGAP9, it lacks the WW domain (Fig. 1a). According to the SMART domain prediction tool [32], bovine, canine and primate ArhGAP9 contain the SH3, WW, PH and RhoGAP domains, murine and rat ArhGAP9 contain the SH3, PH and RhoGAP domains and lack the WW domain. It is possible that genomic deletion resulting in the loss of the WW domain in ArhGAP9 has occurred rodents. To understand the signaling function of the WW domain in ArhGAP9 of organisms that retain this domain, we screened for interacting proteins for the WW domain of human ArhGAP9. The GST fusion protein of the WW domain of human ArhGAP9 immobilized on glutathione Sepharose beads was used as a bait to isolate interacting proteins from rat brain lysate using proteomics (Fig. 1b). Several specific binding proteins were identified, including the MAP kinase Erk2 from bands f and g that generated 15 unique peptides covering 40% of the protein sequence of Mitogen-activated protein kinase 1 (MAPK1, also Erk2). The other bands corresponded to other proteins were characterized separately from this report.

### Binding of Erk2 and p38α to human ArhGAP9 in vitro and in vivo

**Figure 2:**
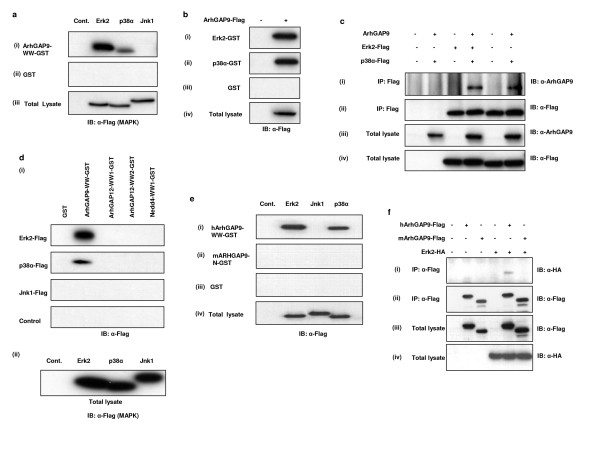
***Erk2 and p38α, but not Jnk1 interacted with ArhGAP9 WW domain***. *a. Specificity of Erk2 and p38α binding to the WW domain of ArhGAP9*. MAP kinases, Erk2-Flag, p38α-Flag or Jnk1-Flag, were transiently expressed in 293T cells and the lysates used for *in vitro *binding assay with (i) ArhGAP9-WW-GST or (ii) GST control, followed by western blotting with α-Flag. (iii) Total cell lysates were separated by SDS-PAGE and immunoblotted with α-Flag. *b. Full length Erk2 and p38α interact with ArhGAP9 in vitro*. Flag-tagged ArhGAP9 was expressed in 293T cells and the lysates were incubated with immobilized (i) Erk2-GST, (ii) p38α-GST or (iii) GST alone as a control. Bound Erk2 and p38α was detected by western blotting with α-Flag. (iv) Total lysates were immunoblotted with α-Flag. *c. Interaction of ArhGAP9 with MAP kinases in vivo*. ArhGAP9 and MAP kinases, Erk2-Flag or p38α-Flag were expressed in 293T cells. The lysates were immunoprecipitated with α-Flag, followed by western blotting with (i) α-ArhGAP9 or (ii) α-Flag. (iii, iv) Immunoblotting of total cell lysates with α-ArhGAP9 or α-Flag. *d. Erk2 and p38α interact specifically with the WW domain of ArhGAP9 but not ArhGAP12 or Nedd4*. MAP kinases Erk2-Flag, p38α-Flag or Jnk1-Flag was expressed in 293T cells and the lysates were incubated with GST fusion proteins of the WW domains of ArhGAP9, ArhGAP12 or Nedd4, or GST alone immobilized on glutathione Sepharose beads, as indicated in the figure. (i) Specifically bound proteins were detected by western blotting with α-Flag. (ii) Total cell lysates were immunoblotted with α-Flag. *e. Erk2 and p38α do not interact with mouse ArhGAP9 in vitro*. Flag-tagged Erk2, p38α or Jnk1 plasmid was expressed in 293T cells. Immobilized GST proteins for (i) WW domain of human ArhGAP9, (ii) N-terminal fragment of mouse ArhGAP9 (residues 1–350) or (iii) GST alone was incubated with the lysates. Bound Erk2, p38α or Jnk1 was detected by western blotting with α-Flag. (iv) Total cell lysates were immunoblotted with α-Flag. *f. Erk2 interact with human but not mouse*. Human or mouse ArhGAP9-Flag was expressed in 293T cells, individually or together with Erk2-HA. The lysates were immunoprecipitated with α-Flag. (i) Bound Erk2 was detected by western blotting with α-HA. (ii) Immunoprecipitated human or mouse ArhGAP9 was detected by α-Flag. Total cell lysates were immunoblotted with (iii) α-Flag or (iv) α-HA.

Erk2 binding to the WW domain of human ArhGAP9 was confirmed by *in vitro *pulldown assays (Fig. 2a). Flag-tagged Erk2 was expressed in 293T cells and the lysates prepared were incubated with GST fusion protein of the WW domain of ArhGAP9 immobilized on glutathione Sepharose beads (ArhGAP9-WW-GST). The binding of two closely related MAP kinases, p38α and Jnk1, was also tested. Consistent with the proteomics data, Erk2 was precipitated by ArhGAP9-WW-GST [Fig. 2a(i)]. While p38α also showed binding albeit to a lesser extent than Erk2, the binding of Jnk1 was not detectable in the pulldown assay. Full-length ArhGAP9 was also shown to be able to bind to immobilized recombinant Erk2 or p38α (Fig. 2b). Using coimmunoprecipitation experiments, the binding of Erk2 or p38α to ArhGAP9 *in vivo *was confirmed [Fig. 2c(i)]. Given that Erk2 and p38α showed no binding to the two WW domains of ArhGAP12 (ArhGAP12-WW1 and ArhGAP12-WW2) or the first WW domain of Nedd4 (Nedd4-WW1) [Fig. 2d(i)], the binding of Erk2 and p38α to the WW domain of ArhGAP9 was specific.

As mouse ArhGAP9 does not contain a WW domain, we tested whether the protein would be able to interact with the MAP kinases Erk2 and p38α like the human ortholog. A GST fusion of an N-terminal fragment of the mouse ArhGAP9 protein (mArhGAP9-N), comprising of amino acids 1–350 which included the SH3 and PH domain and the intervening sequence between these two domains was found to be unable to bind to Erk2 and p38α, when compared to human ArhGAP9 WW domain [Fig. 2e(ii)]. Using coimmunoprecipitation experiments, it was confirmed that full-length mouse ArhGAP9 could not interact with Erk2 *in vivo *(Fig. 2f). Taken together, we conclude that human ArhGAP9 interacted with Erk2 and p38α specifically with its WW domain and the mouse ortholog which lacked a WW domain was unable to bind to MAP kinases.

### MAP kinase and ArhGAP9 binding was mediated by complementarily charged residues in CD and WW domains, respectively

**Figure 3:**
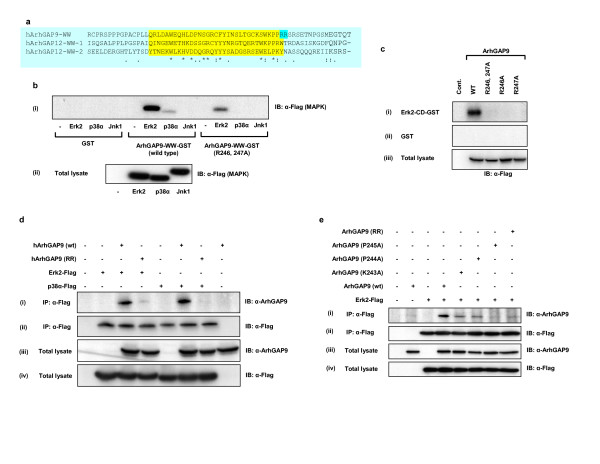
***The ArhGAP9 is a novel MAP kinase docking protein***. *a. Alignment of the WW domains of ArhGAP9 and ArhGAP12*. WW domains of human ArhGAP9 and ArhGAP12 were aligned using ClustalW, highlighted in yellow. The di-Arginine motif at the extreme C terminus of ArhGAP9 WW domain is highlighted in cyan. *b. R246 and R247 in the WW domain of human ArhGAP9 are required for binding to MAP kinases*. Flag-tagged Erk2, p38α or Jnk1 was expressed in 293T cells and the lysates were incubated with immobilized ArhGAP9-WW-GST, ArhGAP9-WW-(R246, 247A)-GST mutant, or GST alone as a control. Specifically MAP kinases were detected by western blotting with α-Flag. (ii) The expression levels of Erk2, p38α and Jnk1 were confirmed to be equivalent by immunoblotting of the total cell lysates with α-Flag. *c. R246 and R247 are required for ArhGAP9 WW domain binding to CD domain of MAP kinase in vitro*. Flag-tagged ArhGAP9 (wild type or mutants R246A, R247A or R246, 247A) was expressed in 293T cells and the lysates were incubated with the (i) Erk2-CD-GST or (ii) GST alone. Bound ArhGAP9 was detected by immunoblotting with α-Flag. (iii) Total cell lysates were immunoblotted with α-Flag. *d. R246 and R247 are required for ArhGAP9 WW domain binding to MAP kinase in vivo*. Flag-tagged Erk2 or p38α was expressed individually or together with full-length ArhGAP9 [wildtype or the R246, 247A mutant (RR)] in 293T cells, as indicated in the figure. The lysates were immunoprecipitated with α-Flag followed by western blotting of the immunocomplexes with (i) α-ArhGAP9 or (ii) α-Flag. Total cell lysates were immunoblotted with (iii) α-ArhGAP9 or (iv) α-Flag. *e. K243, P244 and P245 within the WW domain of ArhGAP9 are also important for MAP kinase binding*. Flag-tagged Erk2 were transiently expressed either individually or together with full-length ArhGAP9 [wildtype or K243A, P244A and P245A mutants] in 293T cells. The lysates were then subjected to immunoprecipitation with α-Flag. The immunocomplexes were resolved by SDS-PAGE followed by western blotting with **(i) **α-ArhGAP9 or **(ii) **α-Flag. Total cell lysates were separated by SDS-PAGE and immunoblotted with **(iii) **α-ArhGAP9 or **(iv) **α-Flag.

Sequence alignment of human ArhGAP9-WW domain with all the known WW domains showed that the C-terminal end of human ArhGAP9-WW domain contained a unique basic di-Arginine motif (R246 and R247) that are not present in all other WW domains compared (data not shown). The alignment of the WW domains of human ArhGAP12 and ArhGAP9 showed that in the first and second WW domains of ArhGAP12 which did not bind Erk2 and p38α, the amino acids in alignment with R247 are hydrophobic residues W and Y, respectively (Fig. 3a). Such short basic motifs, termed Docking (D) domains, had been found to be present in many MAP kinase docking proteins that bind to the conserved docking (CD) domains of MAP kinases [33-36]. The CD domains of Erk2, p38 and Jnk consist of conserved acidic residues which form electrostatic interactions with the basic residues of the target docking proteins [33-36]. Therefore we examined the possibility that the interaction between the complementarily charged residues in the CD domains of Erk2 or p38α and WW domain of ArhGAP9 could be the mechanism which enhanced or mediated the binding of these MAP kinases and ArhGAP9. We investigated whether the basic residues in the WW domain of human ArhGAP9 would mediate the binding to Erk2 and p38α through interaction with the acidic residues in the CD domain, as in the case of other MAP kinase docking proteins. R246 and R247 in the WW domain of human ArhGAP9 were mutated to Alanine (ArhGAP9-WW-RR) and the binding of Erk2, p38α or Jnk1 was tested. As shown in Fig. 3b(i), for both Erk2 and p38α, the binding to the WW domain with R246, 247A mutation was significantly reduced compared to binding to wild type ArhGAP9 WW domain, confirming that these basic residues played a role in MAP kinase interaction. Single mutation of R246A or R247A was also defective in binding to Erk2 (Fig. 3c). The binding of full-length R246, 247A (RR) mutant of ArhGAP9 to Erk2 or p38α was significantly reduced relative to wild type ArhGAP9 *in vivo *using coimmunoprecipitation experiments (Fig. 3d). Therefore, R246 and R247 in the WW domain of ArhGAP9 are important in mediating the binding to Erk and p38 MAP kinases. Further binding analysis indicated that residues in the vicinity of this di-Arginine motif, namely, K243, P244 and P245 with the WW domain also contributed to binding of ArhGAP9 with MAP kinases (Fig. 3e).

**Figure 4:**
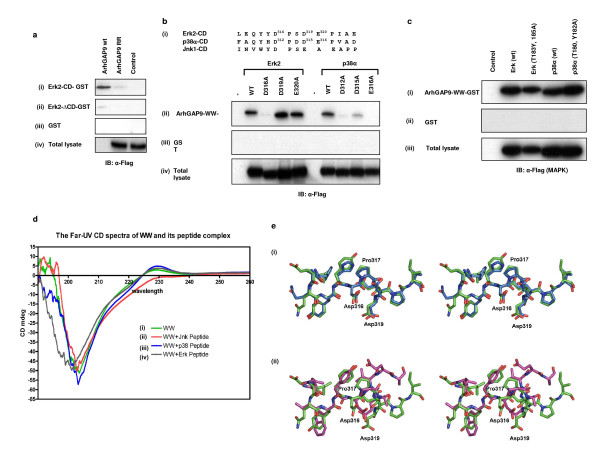
***The Common Docking (CD) domain of MAP kinase mediated the binding to the WW domain of ArhGAP9***. *a*. Flag-tagged full-length ArhGAP9 (wild type or the R246,247A mutant, RR) was expressed 293T cells and the lysates were incubated with GST fusion of (i) the CD domain of Erk2 (Erk2-CD-GST, residues 300–358), (ii) a fragment of Erk2 deleted of the CD domain (Erk2-ΔCD-GST, residues 1–300) or (iii) GST alone as a control. Bound ArhGAP9 was detected by western blotting with α-Flag. (iv) Total cell lysates were immunoblotted with α-Flag. *b. Acidic residues in the CD domain of MAP kinases are important for interaction with ArhGAP9*. (i) Alignment of the Common Docking (CD) domains of Erk2, p38α and Jnk1, the acidic residues mutated to alanine are indicated in superscript. Flag-tagged Erk2 (wild type or D316A, D319A or E320A mutants) or p38α (wild type or D312A, D315A or E316A mutants) was expressed 293T cells. The lysates were incubated with (ii) ArhGAP9-WW-GST or (iii) GST alone. Bound Erk2 or p38α was detected by western blotting with α-Flag. (iv) Total cell lysates were immunoblotted with α-Flag. *c. The activation loop of MAP kinase was not involved in binding with ArhGAP9*. Flag-tagged Erk2 (wild type or T183, Y185A mutant) and p38α (wild type or T180, Y182A mutant) were expressed in 293T cells. The lysates were incubated with immobilized (i) ArhGAP9-WW-GST or (ii) GST alone. Bound Erk2 or p38α was detected by western blotting with α-Flag. (iii) Total cell lysates were immunoblotted with α-Flag. *d. Far-UV CD spectra of WW of ArhGAP9 in complex with MAP kinase CD domain peptides*. (i) The CD spectra of the WW domain of ArhGAP9 indicating that the protein folded properly and has mostly β-sheets and random coils. (ii-iv) CD spectra of ArhGAP9 WW domain in complex with Jnk1, p38α and Erk2 peptides, respectively. Conformational changes were observed for the case of ArhGAP9 WW domain in complex with Erk2 and p38α peptides. As for Jnk1, little effect in the spectra profile was observed when compared with WW domain alone, indicating that no significant binding of the Jnk1 peptide had occurred. *e. Structural alignment of the ArhGAP9-binding regions in the CD domains of Erk2, p38α and Jnk1*. (i) A stereo view of structural alignment of Erk2 (red) with p38α (maroon) (ii) A stereo view of structural alignment of Erk2 with Jnk1. The residues are shown in stick model and the key mutated residues of Erk2 are marked.

To examine whether the CD domains in the MAP kinases Erk2 and p38α were the regions that interacted with the WW domain of ArhGAP9, GST fusions of a C-terminal fragment of Erk2 containing the CD domain (Erk2-CD-GST, residues 300–358) and a deleted version of Erk2 lacking the CD domain (Erk2-ΔCD-GST, residues 1–300) were constructed. The binding of wild type or R246, 247A mutant of ArhGAP9 with immobilized Erk2-CD-GST and Erk2-ΔCD-GST were tested [Fig. 4a(i)]. Wild type ArhGAP9 but not the R246, 247A mutant, specifically precipitated with Erk2-CD-GST, indicating that the CD domain was sufficient to mediate Erk2 binding to ArhGAP9. Consistent with this notion, Erk2-ΔCD-GST did not bind wild type ArhGAP9 [Fig. 4a(ii)].

To further determine whether the conserved acidic residues [Fig. 4b(i)] in the CD domains of the MAP kinases were important in mediating binding to the WW domain of ArhGAP9, point mutants of Erk2 and p38α as indicated in Fig. 4b(ii) were generated and tested for their binding to ArhGAP9. As shown in Fig. 4b(ii), the mutations D316A in Erk2 and D312A, D315A, and E316A in p38α caused a significant reduction in interaction with ArhGAP9-WW domain. These results clearly indicated that the acidic residues in the CD domains of Erk2 and p38α were important for their interactions with ArhGAP9. Other point mutations of Erk2 and p38α including amino acid substitutions in the activation loop of the kinase domain did not affect the binding (Fig. 4c). Further evidence confirming that the WW domain of ArhGAP9 interacted directly with the CD domains of Erk2 and p38 but not Jnk1 was obtained by Far-UV circular dichroism spectroscopy (Fig. 4d). The WW domain is a small three-stranded β-sheet stabilized by the stacking of several conserved aromatic and proline residues [37-40]. Crystal and solution structures of WW domains had shown that WW domains undergo conformational changes upon binding to its peptide ligands [19,20,41]. We applied Far-UV circular dichroism spectroscopy to investigate the conformational changes upon binding of Erk2, p38α or Jnk1 peptides corresponding to the common docking domains to the WW domain of ArhGAP9. The peptides containing 14 amino acids in common docking domains of Erk2, p38α and Jnk1 were synthesized [sequences shown in Experimental procedures and in Fig. 4b(i)]. To analyze the changes in the structure of WW domain upon binding of corresponding peptides, the differences in the Far-UV circular dichroism spectrum of ArhGAP9 WW domain solutions in the presence or absence of the peptides were monitored. The changes in secondary structure contents were analyzed using CDNN program, version 2.1 [42]. The analysis showed that the recombinant ArhGAP9 WW domain folded properly and possessed mostly β-sheets and random coils which are similar to the known WW domains [Fig. 4d(i)]. In the presence of the Erk2 peptide at equimolar concentration, ArhGAP9 WW domain showed significant difference in the circular dichroism spectra particularly from 190–205 nm [Fig. 4d(iv)]. In the presence of p38α peptide, ArhGAP9 WW domain also showed significant difference in the circular dichroism spectra in the same region [Fig. 4d(iii)]. In contrast, the addition of Jnk1 peptide did not alter the circular dichroism spectra significantly although there were notable changes at 220–235 nm [Fig. 4d(ii)]. These conformational changes monitored by Far-UV circular dichroism were in agreement with the other known crystal and solution structures of WW proteins with their peptide targets. This result further supported the conclusion from the *in vitro *and *in vivo *experiments that both Erk2 and p38α interacted with ArhGAP9-WW domain but Jnk1 did not. However, the exact conformational changes and mechanism of binding can be obtained only by solving the crystal or solution structures of ArhGAP9-WW domain in complex with its binding partners. The data described above strongly indicated that the binding between ArhGAP9 and the MAP kinases Erk and p38 is specific and is mediated by the WW and CD domains of ArhGAP9 and the MAP kinases, respectively.

To gain insights into the structural basis of specificity of the WW domain of ArhGAP9 binding to Erk2, p38α and Jnk1, we compared the three-dimensional structures of CD domains of the three proteins. It could be seen from the comparison that although the overall structures of Erk2 (PDB id: 1ERK), p38α (PDB id: 1P38) and Jnk1 (PDB id: 1JNK) are very similar, the CD domain of p38α shared greater homology than that of Jnk1 to Erk2. Furthermore while Erk2 and p38α are significantly super-imposable with RMSD of 0.68 Å [Fig. 4e(i)], Erk2 and Jnk1 CD domains are relatively distinct with RMSD of 2.18 Å [Fig. 4e(ii)]. The high sequence and structural similarity between Erk2 and p38α provided some insights into the structural basis for the specificity on the WW domain of ArhGAP9 for Erk2 and p38α but not Jnk1, as observed in the binding studies *in vitro *and *in vivo*.

### Coexpression of MAP kinase kinases (MAPKKs) reduced binding of MAP kinases (MAPKs) to WW domain of ArhGAP9

**Figure 5:**
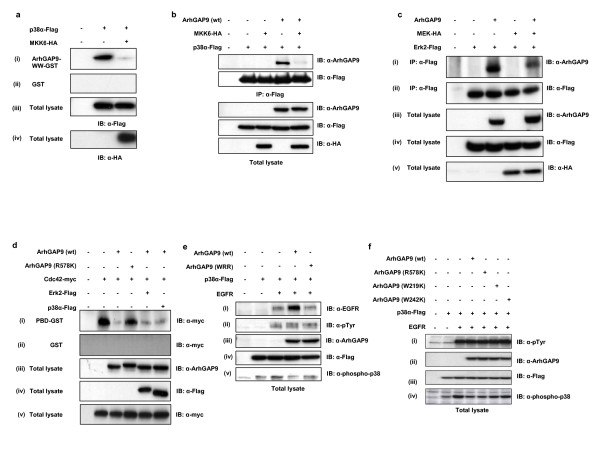
***ArhGAP competes with MAP kinase kinases (MAPKKs) to suppress MAP kinase activation***. *a. Coexpression of MKK6 dimished the binding of p38α to the WW domain of ArhGAP9*. p38α-Flag was transfected alone or together with HA-tagged activated mutant of MKK6 in 293T cells and the lysates were incubated with (i) ArhGAP9-WW-GST or (ii) GST alone as a control. Bound p38α was detected by western blotting with α-Flag. (iii, iv) Total cell lysates were immunoblotted with α-Flag or α-HA, respectively. *b. Overexpression of MKK6 diminished the binding between ArhGAP9 and p38α*. Flag-tagged p38α, HA-tagged MKK6 and ArhGAP9 were expressed in 293T cells as indicated in the figure. The immunocomplex of p38α was analyzed by western blotting with (i) α-ArhGAP9 and (ii) α-Flag. (iii-v) Total lysates were immunoblotted with α-ArhAGAP9, α-Flag and α-HA, respectively. *c. Reduction of ArhGAP9 binding to Erk2 by MEKoverexpression in vivo*. Flag-tagged Erk2, HA-tagged MEK2 and ArhGAP9 were transiently transfected in 293T cells as indicated in the figure. Immunoprecipitation was carried out with α-ArhGAP9, followed by western blotting with (i) α-ArhGAP9 α-Flag and (ii) α-HA. Western blotting of the total lysates with (iii) α-phospho-Erk showed that Erk2 was activated when coexpressed with MEK2. Western blotting of the total lysates with (iv) α-ArhAGP9, (v) α-Flag and (vi) α-HA showed the expression of ArhGAP9, Erk2 and MEK2 in the total cell lysates. *d. MAP kinase binding has no significant effect on ArhGAP9 RhoGAP activity*. ArhGAP9 (wild type or the GAP-inactive mutant, R578K) was transfected with myc-tagged Cdc42, as well as Flag-tagged Erk2 or p38α in 293T cells, as indicated in the figure. (i) The lysates were incubated with immobilized PBD-GST followed by detection of the relative amounts of active cdc42 precipitated by western blotting with α-myc. (ii) GST alone was used as a control. (iii-v) Total cell lysates were immunoblotted with α-ArhGAP, α-Flag or α-myc, rspectively. *e. ArhGAP9 binding suppresses MAP kinase activation by EGFR*. ArhGAP9 [wild type or the triple mutant W242K, R246A, R247A (WRR)] was expressed in 293T cells alone or with EGFR and Flag-tagged p38α, as indicated in the figure. (i-v) Total cell lysates were immunoblotted with antibodies to EGFR, phosphotyrosine, ArhGAP9, Flag epitope or phospho-p38α. *f. RhoGAP activity of ArhGAP9 is not required for its suppression of MAP kinase activation*. Wild type ArhGAP9 or the mutants R578K (GAP-inactive), WW-1 (W219K) and WW-2 (W242K) were cotransfected with EGFR and p38α in 293T cells. (i-iv) Total cell lysates were immunoblotted with antibodies to phosphotyrosine, ArhGAP9, Flag epitope or phospho-p38α.

The CD domains of MAP kinases had been established as the binding sites for their upstream activating kinases, namely MEK1 and 2 for Erk1 and 2, and MKK3, 4 and 6 for p38α. [33,35,36,43]. Some other regulators, effectors as well as MAP kinase substrates have also been established to dock on the CD domains. These docking proteins all contain positively charged residues that form the docking (D) domain for binding with the CD domain of MAP kinases [23,25-27]. From our data that the binding of ArhGAP9 to Erk2 and p38α required a di-Arginine motif in the WW domain and conserved acidic residues in the CD domain, it was possible that the binding of D domain containing proteins such as MEK2 and MKK6 may compete with the binding of ArhGAP9 to Erk2 and p38α, respectively. We therefore compared the binding of p38α to ArhGAP9-WW domain in the absence or presence of overexpressed MKK6 [Fig. 5a(i)]. The amount of p38α that precipitated with the WW domain ArhGAP9 was significantly reduced in the presence of MKK6, indicating that it was possible that the binding of MKK6 to p38α could inhibit the binding of the WW domain ArhGAP9. Consistent with the above *in vitro *data, the binding of full-length ArhGAP9 to p38α *in vivo *was reduced in the presence of MKK6 (Fig. 5b). We had also observed that a mutant of MKK6 with the basic residues in the D domain responsible for binding to the CD domain of p38α deleted failed to bind p38α and therefore to inhibit p38α binding to the WW domain ArhGAP9 (data not shown). Similarly, with the cotransfection of MEK2, the amount of ArhGAP9 that co-immunoprecipitated with Erk2 was significantly reduced (Fig. 5c). Taken together, these results indicated that upstream activating kinases of p38α and Erk2, namely MKK6 and MEK2, respectively were able to disrupt the binding of the MAP kinases to the WW domain of ArhGAP9, most likely due to their docking onto the CD domains and competitively preventing the binding of ArhGAP9.

### Binding of ArhGAP9 inhibited Erk2 and p38α activation by upstream signals

Given that both ArhGAP9 and the MAP kinases possess catalytic activities, we addressed the question of whether their interaction would influence their activities. Furukawa *et al *[7,17] as well as our data unpublished showed that ArhGAP9 functioned as an active GTPase activating protein (GAP) towards cdc42 and Rac1 but not RhoA. To determine whether MAP kinase binding had any effect on the RhoGAP activity of ArhGAP9, we compared the levels of active cdc42 in the lysates of cells transfected with ArhGAP9 in the presence or absence of Erk2 or p38α using the Pak-binding domain (PBD) GST method (Fig. 5d). Through protein sequence alignment of the RhoGAP domain of human ArhGAP9 and other known RhoGAPs whose activities and structures have been determined, we identified R578 in human ArhGAP9 as the 'Arginine finger' residue essential for GAP activity. We mutated R578 was to lysine and confirmed that ArhGAP9 (R578K) was catalytically inactive by *in vitro *GAP assays (data not shown). Using the GAP-inactive mutant of ArhGAP9 (R578K) as a negative control for the assay, there was clearly no significant effect in the activity with or without the presence of Erk2 or p38α.

We next investigated whether the binding of ArhGAP9 to the Erk2 and p38α had an effect on the MAP kinase activities. 293T cells were transfected with p38α-Flag alone, or p38α-Flag with EGFR to induce the activation of p38α, or p38α with EGFR as well as ArhGAP9, either wild type or the W242K, R246, 247A (WRR) triple mutant that was defective in binding to Erk and p38, as indicated in Fig. 5e. As shown by western blotting with α-phospho-p38 in Fig. 5e(v), p38α transfected alone showed a slight detectable level of basal activation which increased significantly with the coexpression of EGFR. With the expression of wild type ArhGAP9, a reduction of p38α activation induced by EGFR was observed, indicating that ArhGAP9 could suppress the activation of p38α downstream of EGFR. With coexpression of the ArhGAP9 (WRR) mutant, there was a slighter suppression of p38α activation by EGFR indicating that this mutant was able to partially restore p38α activation by EGFR inhibited by wild type ArhGAP9. This result strongly suggested that the binding of ArhGAP9 to MAP kinases prevented the activation of the latter by their upstream kinases.

We further confirmed that this inhibitory effect was not attributed to the RhoGAP activity but to the WW domain of ArhGAP9. 293T cells were transfected with p38α-Flag alone, or p38α-Flag with EGFR, or p38α with EGFR as well as ArhGAP9, either wild type, the GAP-inactive mutant (R578K) or the WW domain mutants (W219K or W242K), as indicated in Fig. 5f. As shown in Fig. 5f(iv), the suppressive effect of wild type ArhGAP9 on EGFR-induced p38 activation was not affected by the R578K mutation, but was partially reversed by the W219K or W242K mutation.

Taken together, the above data demonstrated that (a) the binding of ArhGAP9 to MAP kinases through its WW domain caused the inhibition of MAP kinase activation by upstream signals and (b) this interaction did not have a significant effect on the RhoGAP activity of ArhGAP9. (c) In addition, the inhibitory effect of ArhGAP9 on MAP kinase activation was independent of its RhoGAP activity.

### ArhGAP9 binding prevented MAP kinase-induced loss of actin stress fibres in fibroblasts

**Figure 6:**
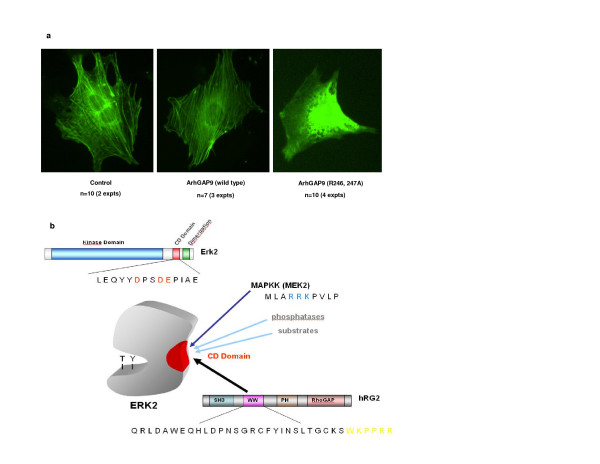
*a. Expression of ArhGAP9 R246, 247A mutant disrupts stress fibres in Swiss 3T3 fibroblasts*. Swiss 3T3 cells were microinjected with full-length ArhGAP9 (wild type or the R246, 247A (RR) mutant) together with GFP-actin. The cells were imaged for GFP fluorescence. *b. Proposed mechanism of negative regulation of MAP kinase by ArhGAP9*. ArhGAP9 contains a WW domain which possesses a basic di-Arginine motif while MAP kinase (MAPK) contains a Common Docking (CD) domain that contains conserved acidic residues. In quiescent state, ArhGAP9 interacts with MAPK through electrostatic interaction between the complementary basic and acidic residues in the WW and CD domains, respectively thus blocking the access of MAPK by other docking proteins and negatively regulating MAPK activation. In the induced state, the presumable increase in local concentration of active upstream MAPK kinase (MAPKK) displaces ArhGAP9 by docking onto CD domain of MAPK, causing the diminished binding of ArhGAP9 to MAPK. The interaction between MAPK and MAPKK results in the phosphorylation of MAPK in the kinase activation loop to activate the latter.

Rho GTPase and MAP kinase signaling have both been implicated in the control of the actin cytoskeleton. Specifically it was reported that activation of the MAP kinases Erk1, 2 and 5 pathways caused disruption of the actin cytoskeleton [44]. To investigate if the interaction of ArhGAP9 and MAP kinases had an effect on the actin cytoskeleton, ArhGAP9 (wild type or R246, 247A mutant) and GFP-actin were heterologously expressed in Swiss 3T3 cells by microinjection of the respective cDNAs, followed by live imaging of the actin structure. As shown in Fig. 6a, in the presence of wild type ArhGAP9, the cells possessed distinct bundles of actin stress fibres as in the control cells where GFP-actin alone was expressed. However, in the presence of the R246, 247A ArhGAP9 mutant, the stress fibres dissolved and showed a diffuse cytosolic pattern of actin distribution. These results supported the hypothesis that binding of wild type ArhGAP9 to MAP kinases sequestered them in the inactive forms, preventing the activation of the MAP kinases and therefore allowing the formation and maintenance of actin stress fibres in fibroblasts. Conversely, the R246, 247A mutant being unable to bind the MAP kinases, would potentially allow activation of the MAP kinases to result in the disassembly of the actin stress fibres.

## Discussion

ArhGAP9 is a recently identified RhoGAP which contains four functional domains including SH3, WW, PH and RhoGAP. Interestingly, the rodent orthologs do not contain the WW domain despite sharing more than 60% identity with the canine, bovine and primate proteins. We focused on studying the role(s) of the WW domain in human ArhGAP9 and demonstrated that it possesses a unique ligand specificity. Using proteomics and mass spectrometry we found that the WW domain of the human ArhGAP9 associated specifically with the MAP kinase, Erk2. The WW domain of ArhGAP9 also bound to another member of the MAP kinase family, p38α, albeit with an apparently less affinity but not to Jnk1. The Erk2 and p38α proteins do not contain any PPDY, PPLP, PPPPP, PPXPPXR, PPRXXP, PR motifs or (pT/pS)-P determined for WW domain binding, indicating that the WW domain of ArhGAP9 had a novel ligand specificity distinct from the four groups currently established. We further showed that the binding of the WW domain of ArhGAP9 with Erk2 or p38α was mediated through complementarily charged residues between the C-terminal end of the WW domain of ArhGAP9 and the conserved Common Docking (CD) domain in Erk2 or p38α and proposed that ArhGAP9 is a novel Erk2 and p38α docking protein. The binding between the WW domain of human ArhGAP9 and the MAP kinases is most likely not solely mediated by the electrostatic interactions between the di-Arginine motif in the WW domain of ArhGAP9 and the acidic residues in the CD domain of the MAP kinases. These interactions may however be important in providing electrostatic steering forces to control the rate of association or specificity of binding. The three-dimensional structure of Erk2 shows that the CD domain was located on the opposite surface of Erk2 from the activation loop, supporting our data that the activation loop region was not involved in binding to the WW domain of ArhGAP9. In this study, we have identified that a di-Arginine motif (R246 and R247) in the C-terminal region of the WW domain of ArhGAP9 was important for the binding to MAP kinases as the mutation of these basic residues either individually or together abolished the binding of full-length ArhGAP9 or the WW domain alone to Erk2 or p38α. We have also mapped the acidic residues in the CD domains of Erk2 and 38α required for binding to the WW domain of ArhGAP9. The residues D316 in Erk2 and D312, D315, and E316 in p38α have been found to be important for binding to ArhGAP9. While it is clear that the di-Arginine motif in the C-terminal region of the WW domain as well as the two signature tryptophan residues were important for binding to Erk2 and p38α, the overall structural mechanism of MAP kinases binding to the WW domain of ArhGAP9 and the other residues on the WW domain and the MAP kinases that might also contribute to the binding specificity and affinity remains to be determined. The determination of the three-dimensional structure of the ArhGAP9 WW domain in a complex with Erk2 or p38α will provide further evidence on the contribution of other regions of the WW domain and MAP kinases in the interaction.

The binding of human ArhGAP9 and the MAP kinases Erk2 and p38α represents another example of the recently discovered cross-talk mechanism between the Rho GTPase and MAP kinase signaling pathways. There are 2 recent reports of examples of RhoGAPs interacting with Erk, namely IQGAP1 and CdGAP. In the case of IQGAP1, Erk2 binds to the WW domain of IQGAP1 although the specific residues involved had not been elucidated. The overexpression or reduction of IQGAP1 expression by siRNA both caused the activation of Erk2 by EGF or IGF-1 in MCF-7 cells to be reduced, probably due to an optimal level of intracellular IQGAP1 being necessary for maximal level of activation of Erk by EGF or IGF-1 [45]. The WW domain of IQGAP1 does not contain basic residues analogous to R246 and R247 in ArhGAP9 and we had found that it bound Erk2 with a much lower affinity that ArhGAP9 (data not shown). In the case of CdGAP, Erk1 binds to the RhoGAP through a putative DEF (Docking for Erk FXFP), resulting in phosphorylation of the RhoGAP by the MAP kinase and inhibition of its RhoGAP activity [46]. CdGAP does not contain a WW domain and is therefore expected to associate with Erk through a different docking mechanism.

The RhoGAP activity of ArhGAP9 was not significantly altered as a result of the interaction with MAP kinases but rather, the activation of the MAP kinases was suppressed by the interaction. The binding of Erk2 or p38α to ArhGAP9 was significantly reduced in the presence of MAP kinase docking proteins such as upstream activating kinases, namely MEK2 for Erk2 or MKK6 for p38α. We hypothesized that in the quiescent state, ArhGAP9 may bind to Erk2 or p38α in the cell through its WW domain docking on the CD domain of the MAP kinases. When upstream signals were elicited to activate the MAP kinases, the binding of ArhGAP9 was diminished probably due to competitive displacement by other docking proteins, namely the MAP kinase kinases, MEK for Erk and MKK3, 4 or 6 for p38. The subcellular localization and concentrations of the docking proteins are most likely key determining factors of the docking interactions with MAP kinases. We have proposed a model for the negative regulation of MAP kinase signaling by ArhGAP9 as illustrated in Fig. 6b. Consistent with this hypothesis, our data showed that while wild type ArhGAP9 significantly suppressed Erk2 or p38α activity induced by EGFR, the mutants of ArhGAP9 that was defective in binding to the MAP kinases resulted in only marginal suppression of EGFR-induced MAP kinase activation.

Both the Erk and p38 MAP kinases play pivotal signaling roles in development and cellular processes, including cytoskeletal changes that underlie increased cell motility and decreased cell adhesion in transformation and metastasis. In almost all types of cancers, the Erk MAP kinase is constitutively active, conferring increased proliferation and resistance to apoptotic stimuli, including chemical cytotoxic drugs. The Erk MAP kinase is therefore a highly relevant target for therapeutic intervention in cancer. Extensive published literature has demonstrated that the Ras/Raf/Erk pathway downstream of *ras *or *src *oncogenes was required for disruption of actin stress fibres and focal adhesions in different transformed cell lines, indicating the importance of Erk 1 and 2 activity in actin cytoskeleton remodeling [44,47-49]. Activation of Erk in transformed cells has been shown to cause the down-regulation of Rho-kinase expression [49] and activation of LIM kinase [50,51]. Rho-kinase phosphorylates myosin light chains and negatively regulates myosin phosphatase, which result in increased acto-myosin-based contractility [52,53] which contribute to the formation of stress fibres and focal contacts. LIM kinase phosphorylates cofilin to result in the inhibition of its actin-depolymerization activity thus contributing to actin fibre stabilization [50,51]. Consistent with these reported roles of Erk activity in the control of the actin cytokeleton, our results showed that expression of wild type ArhGAP9 preserved the stress fibres in fibroblasts. In contrast, expression of the R246, 247A mutant defective in binding to Erk caused dramatic disassembly of actin stress fibres, with the unpolymerized actin distributed to the cytosol.

Our data collectively demonstrated that ArhGAP9 is a novel docking protein of the MAP kinases Erk and p38, but not Jnk. Through a series of biochemical, biophysical and cell-based experiments, we have provided evidence that the activation of the MAP kinases by upstream activating kinases was suppressed by ArhGAP9 binding both *in vitro *and *in vivo*. This provides a basis for a previously unexplored molecular mechanism to modulate MAP kinase activity and could be explored further for development of novel inhibitors of MAP kinase signaling by disruption of MAP kinase binding to docking proteins at the CD domain. This strategy should allow higher selectivity in targeting MAP kinases compared to the more general ATP-binding interference approach. Indeed, using the computer-aided drug design (CADD) approach, novel compounds that disrupt the interactions between Erk and their docking proteins through the CD and D domains have been identified and shown to be effective in blocking Erk activation in cells [34,54-56].

## Materials and methods

### Antibodies

α-Flag (M2) and α-phospho-p38 were from Sigma, α-phospho-Erk was from Cell Signaling Technology, α-HA (12CA5) was from Boerhinger, α-ArhGAP9 was generated by standard immunization procedure with full-length human ArhGAP9-GST as the immunogen. α-phosphotyrosine conjugated to horse radish peroxidase (PY20-HRP) was from Transduction Laboratories, α-EGFR was from Santa Cruz Biotechnology.

### Cell lines

293T cells were cultured in Dulbecco's minimal essential medium (DMEM) containing 4.5 g/l D-glucose, 10% (v/v) fetal bovine serum (HyClone), 10 mM L-glutamine, and 100 μg each of penicillin and streptomycin/ml from Sigma. Swiss 3T3 were cultured in DMEM containing 4.5 g/l D-glucose, 10% (v/v) Cosmic calf serum (HyClone), 10 mM L-glutamine, and 100 μg each of penicillin and streptomycin/ml.

### Plasmids

Human ArhGAP9 (wild type and mutants) and EGFR coding cDNAs were cloned into the mammalian expression vector pRK5. Erk2, p38, Jnk1 cDNAs were cloned in pxJ40-Flag vector with an N-terminal Flag tag. HA-tagged activated MEK2(S222D, S226D) was cloned in pUSEamp vector. Activated MKK6(S207E, T211E) was cloned in pxJ40-HA vector. Plasmid encoding Pak-binding domain (PBD)-GST was obtained from Dr. E Manser (CMM, A-STAR, Singapore). Cdc42-myc was cloned in pcDNA3. For transient expression in mammalian cells, Lipofectamine (Gibco BRL) was used for transfection, following the protocol recommended by the manufacturer. Point mutations were introduced with the Quikchange site-directed mutagenesis kit (Stratagene) and the mutations were confirmed by DNA sequencing.

### Protein expression and purification

For expression of glutathione S-transferase (GST) fusion proteins, cDNA sequences were cloned into the pGEX4T1 vector (Pharmacia). The GST fusion proteins were expressed in E. coli BL21 (DE3) cells and purified using glutathione sepharose 4B column chromatography (Amersham). For Far-UV CD spectroscopy, the GST tag was cleaved with the thrombin protease by overnight on-column digestion at 4°C. The resulting protein preparation was then further purified by using ion-exchange Mono Q Sepharose column (Amersham) which had been pre-equilibrated with buffer A (20 mM Tris-HCl pH 7.5). The protein was then eluted from the column with a linear gradient to buffer B (20 mM Tris-HCl pH 7.5, 1 M NaCl). The protein was further purified using Superdex-75 gel filtration column chromatography in Biologic Duoflow FPLC system.

### Proteomics and mass spectrometry analysis

Protein complexes of the WW domain of human ArhGAP9 isolated from rat brain lysate were eluted and resolved by 10% SDS-PAGE, followed by detection of bound proteins by staining with Colloidal Coomassie Blue (Pierce). Specific bands were excised and subjected to in-gel reduction, S-alkylation and trypsin hydrolysis. Liquid-chromatography tandem mass spectrometry (LC-MS/MS) analysis of the peptides was performed on a Finnigan LCQ Deca ion trap mass spectrometer (Thermo Finnigan) fitted with a Nanospray source (MDS Proteomics). Chromatographic separation was conducted using a Famos autosampler and an Ultimate gradient system (LC Packings) over Zorbax SB-C18 reverse phase resin (Agilent) packed into 75 μm PicoFrit columns (New Objective). Protein identifications were made using the search engines Mascot (Matrix Sciences) and Sonar (ProteoMetrics).

### In vitro binding assays

Lysates for binding assays and immuno-precipitation were prepared by lysis of cells in the cell lysis buffer [20 mM HEPES (pH 7.5), 137 mM NaCl, 1% Triton X-100, 10% glycerol, 1.5 mM MgCl_2_, 1 mM EGTA, 0.1 mM Na_3_VO_4 _and Complete Protease inhibitors (Boehringer)], followed by centrifugation 13,000 *g *for 15 min at 4°C and collecting the supernatant. For *in vitro *pulldown assays, GST fusion proteins immobilized on glutathione beads were incubated with the lysates for 1 hour at 4°C with rotation, followed by washing of the beads with specifically bound proteins with cell lysis buffer for 5 times, each time for 5 min with rotation at 4°C. The protein complexes were then eluted with Laemmli Buffer and resolved by sodium dodecyl sulfate-polyacrylamide gel electrophoresis (SDS-PAGE), followed by blotting onto PVDF membranes and detection with specific antibodies. For immuno-precipitation, antibodies were added to the lysates and incubated for 1 hour at 4°C with rotation, followed by addition of Protein A PLUS G beads (Calbiochem) to capture the immuno-complexes. The immuno-complexes were washed and then eluted with Laemmli Buffer and resolved by SDS-PAGE, followed by western blotting with specific antibodies.

### Peptides and Far UV Circular Dichroism (CD) spectroscopy

Peptides for Erk2: LEQYYDPSDEPIAE, p38α: FAQYHDPDEPVAD and Jnk1: INVWYDPSEAEAPP were custom synthesized by NeoSystems (Strasbourg, France). CD experiments were performed with a Jasco J-715 spectropolarimeter equipped with a Peltier cell holder and a PTC-348WI temperature controller. Far-UV CD spectra were measured in a 1-mm rectangular quartz cell. The buffer used was 20 mM Tris-HCl (pH 7.5), 500 mM NaCl and 1 mM DTT. Protein concentration of approximately 0.5 mg/ml determined by the Bradford assay method was used for wavelength scans. Wavelength scans were made at a scan rate of 10 nm/min and the average value of 3 scans of the same solution were obtained. Data were collected at 25°C over a wavelength range 190–260 nm with a bandwidth of 1 nm. To determine the conformational changes in WW domain upon peptide binding, the ellipticity of the corresponding peptides at equimolar concentration with the solvent were subtracted from the ellipticity of the WW domain-peptide complex and similarly the solvent spectrum were subtracted from the spectrum of WW domain before analysis. The far UV CD spectra were analyzed by using the secondary structure analysis program CDNN, version 2.1 [42].

### Microinjection and live cell imaging

Swiss 3T3 fibroblasts were plated at 5 × 10^4 ^per glass bottom dish and grown overnight at 37°C in DMEM with 10% Cosmic calf serum. DNA was injected at 0.5 μg/ml into the nuclei using a custom setup microinjector and Olympus microscope (Olympus IMT-2) and the cells were returned to incubation at 37°C overnight to allow for protein expression. For DIC and fluorescence time-lapse analysis, cells were incubated on a heated stage at 37°C and imaged with a monochromator on a Zeiss Axiovert 200 M microscope enclosed in an incubator with CoolSNAP CCD camera.

## Competing interests

The author(s) declare that they have no competing interests.
